# Case report: The first case of Mpox in a patient with HIV in Burundi

**DOI:** 10.3389/fmed.2025.1536774

**Published:** 2025-02-25

**Authors:** Doriane Sabushimike, Marc Nimburanira, Esther Freeman, Déo Simbarariye, Armel Nzeyimana, Wendemagegn Enbiale

**Affiliations:** ^1^Unit of Dermatology, Department of Internal Medicine, Kamenge Military Hospital, Bujumbura, Burundi; ^2^Department of Internal Medicine, Kamenge Military Hospital, Bujumbura, Burundi; ^3^Harvard University, Cambridge, MA, United States; ^4^Department of Dermatology, Massachusetts General Hospital, Boston, MA, United States; ^5^Bujumbura Center Health District, Bujumbura, Burundi; ^6^Department of Dermatology, College of Health Sciences and Medicine, Bahir Dar University, Bahir Dar, Ethiopia

**Keywords:** Mpox, HIV, coinfection, investigation, complications

## Abstract

**Introduction:**

Mpox is a viral disease that primarily affects individuals living in endemic regions. The 2022 outbreak notably impacted HIV-positive individuals, who were disproportionately affected. This report describes the first confirmed case of Mpox in Burundi, involving an HIV-positive patient with advanced disease. The case was confirmed by the national laboratory during the early stages of the 2024 outbreak.

**Case presentation:**

A 50-year-old woman presented with pustular and necrotic skin lesions, vulval ulcers, and systemic symptoms. She was diagnosed with HIV upon initial evaluation and, despite an initial misdiagnosis, was ultimately confirmed to have a co-infection of Mpox. Complications included secondary bacterial infection, severe pain, and prolonged healing.

**Conclusion:**

HIV coinfection can exacerbate Mpox, leading to severe clinical presentations. Therefore, early recognition and screening for HIV in Mpox patients are essential.

## Introduction

Mpox is an illness caused by a virus from the Poxviridae family, which consists of double-stranded DNA viruses capable of infecting both humans and animals (1). A related virus, variola, responsible for smallpox, was successfully eradicated in the 1980s (2).

Historically, Mpox has been found in certain regions of West and Central Africa (3, 4). However, in May 2022, a sharp rise in cases outside these endemic areas prompted the World Health Organization to declare the outbreak a Global Public Health Emergency on 23 July 2022 (5). In Burundi, the Ministry declared an outbreak on 25 July 2024.

The classic clinical manifestations of Mpox often include fever, headache, myalgia, lymphadenopathy, and skin lesions that progress through papular, vesicular, and pustular stages before ulcerating and crusting. The typical pustules of Mpox are umbilicated on an erythematous base and can occur on the face, palms, soles, and genital areas. Severe cases may include complications such as secondary bacterial infections, severe ulcerations, and prolonged illness (1).

Diagnostic confirmation is primarily achieved through molecular DNA amplification techniques applied to samples collected from skin lesions using a cutaneous swab. Polymerase chain reaction (PCR) is commonly performed, followed by clade-specific testing (6). Histology can be performed, and its utility lies in supporting the diagnosis when molecular tests are not available or in cases of atypical presentations. The histological examination reveals hyperkeratosis, acanthosis, and ballooning degeneration of keratinocytes, often accompanied by viral cytopathic effects (7).

During the 2022 outbreak, individuals with HIV were disproportionately affected, accounting for 38–50% of Mpox diagnoses (8). The majority of people living with HIV reported in the 2022 global case series had HIV viral suppression, with median CD4 counts of more than 500 cells per mm (3). Their clinical presentations, time to Mpox viral clearance, and outcomes were similar to those of people without HIV (9, 10).

This report presents the first case of Mpox in Burundi during the 2024 outbreak, involving a 50-year-old HIV-positive woman, and highlights the need for early diagnosis and intervention in such cases.

## Case presentation

### History

A 50-year-old woman presented to the emergency department of the Kamenge Military Hospital in Bujumbura, Burundi, with a 1-week history of fever, general body weakness, and skin lesions on her hands and genitalia. The patient reported no prior medical history or did not use any chronic medications. Initial blood work revealed leukocytosis with neutrophil predominance. On admission, HIV testing was positive, confirming advanced-stage HIV (Stage 4). She was subsequently admitted to the internal medicine department for further evaluation and treatment of suspected sexually transmitted infections (STIs).

**Figure 1:**
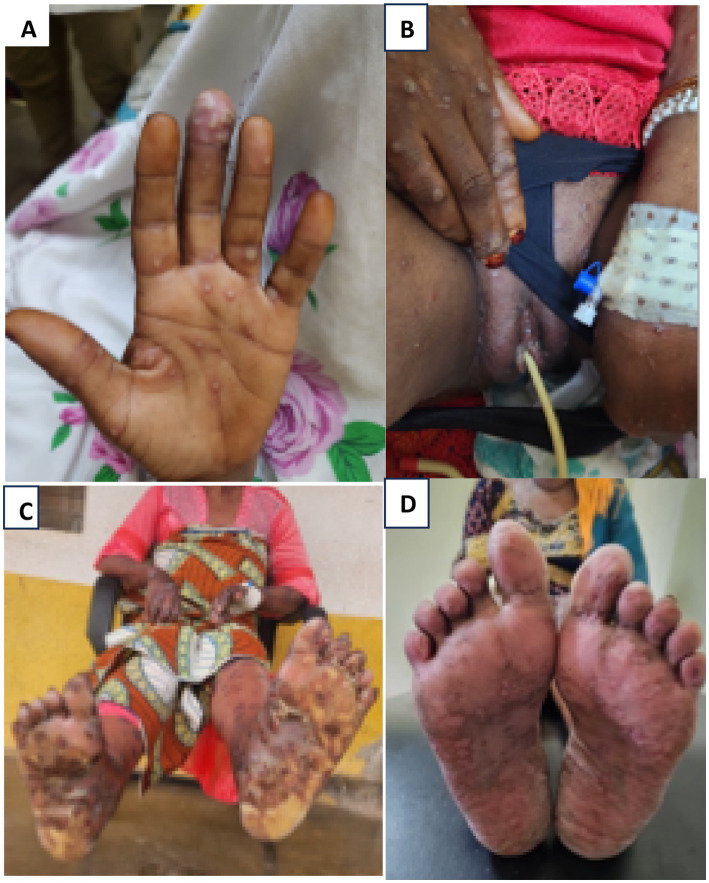
**(A)** Umbilicate papules and pustules on hands. **(B)** Edematous vulva and ulcers on labia minora. **(C)** Necrosing ulcers. **(D)** 1month post discharge.

During her admission, a dermatology consultation was requested due to the progression of her skin lesions. The dermatological examination revealed pustules on both hands (Figure 1A) and painful, umbilicated, excoriated papules on an erythematous base on her face and arms. In addition, the vulva was swollen with shallow ulcers on the major labia (Figure 1B), accompanied by pain and purulent discharge. Mpox was suspected.

### Investigation

Confirmatory testing for Mpox was performed using PCR with a cutaneous swab at the national laboratory. A full blood count (FBC) was also performed, revealing leukocytosis with a predominance of neutrophils.

### Management

Due to her fever, pain, genital discharge, and elevated white blood cell count, intravenous ceftriaxone (1 g twice daily) and paracetamol (1 g thrice daily) were initiated. Over the following days, the lesions spread to other parts of her body, accompanied by worsening vulval discharge, severe pain, and new necrotic lesions that emitted a foul odor. Intravenous metronidazole (500 mg thrice daily) was added to her treatment regimen to target anaerobic and Gram-negative bacteria.

Despite these measures, the patient’s condition deteriorated further during the second week, with persistent fever, myalgia, anorexia, sore throat, oral candidiasis, and clinical signs of septicemia. Due to persistent signs of systemic infection and clinical signs of septicemia in week 2, such as fever, tachycardia, and slow pulse, and the lack of access to blood cultures, ceftriaxone and metronidazole were discontinued and intravenous levofloxacin (500 mg daily) was initiated.

The wound care involved potassium permanganate baths for the vulvar ulcers and necrotic lesions on other parts of the body, along with mupirocin 2% ointment to treat localized bacterial infections. For pain relief, the patient was prescribed tramadol (50 mg twice daily) in addition to paracetamol. Antifungal therapy with nystatin (10,000 IU, four drops every 6 h) was introduced after the development of oral candidiasis. Cotrimoxazole (860 mg once daily for one month) was also administered to provide prophylaxis against opportunistic infections.

Gradual clinical improvement was observed after a week of levofloxacin, at which point it was discontinued. The facial lesions healed completely, leaving post-inflammatory hyperpigmentation. However, the pustules on the feet coalesced into large bullae with necrotic ulcers that emitted a foul odor and attracted flies (Figure 1C). To manage this, vinegar soaks were performed twice daily, followed by mupirocin application, which led to visible healing and re-epithelialization within 1 week.

### Outcome

The patient was hospitalized for 2 months, achieving complete re-epithelialization and recovery. Upon discharge, she was clinically stable and initiated on antiretroviral therapy (ART). A follow-up one month later showed complete healing with residual post-inflammatory hyperpigmentation (Figure 1D).

## Discussion

This case highlights the atypical presentation of Mpox in an HIV-positive patient with advanced disease that led to a delay in diagnosis.

Although some reports during the early Mpox outbreak in 2022 described the self-limiting clinical course in individuals with well-controlled HIV as being very similar to that in individuals without HIV, a global case series published in 2023 provided evidence of a more severe presentation among people with HIV and low CD4 cell counts (CD4 < 350 cells per mm^3^) (8).

Data from Nigeria and the USA suggest poor clinical outcomes in people with more HIV-related immunosuppression. Two reports from Nigeria during the 2017–2018 outbreak suggested a higher mortality rate among untreated HIV patients, along with more confluent rashes, higher rates of secondary bacterial infections, and more prolonged illness, compared to people without HIV (4, 11). During the 2022 outbreak in the USA, the US Centers for Disease Control and Prevention (CDC) supported the findings from Nigeria, reporting 47 cases of severe Mpox among people with advanced, uncontrolled HIV infection. All 47 individuals were hospitalized, had prolonged disease courses, and developed complications, and five deaths were attributed to Mpox (12). More severe rectal diseases, such as rectal bleeding and purulent or bloody stools, have been observed in individuals with Mpox and HIV, particularly among those with unsuppressed viral loads or lower CD4 counts. In the CDC series, individuals with HIV and Mpox were more likely to experience rectal pain (34 vs. 26%), tenesmus (20 vs. 12%), rectal bleeding (19 vs. 12%), purulent or bloody stools (15 vs. 8%), and proctitis (13 vs. 7%) compared to those without HIV. Among those with unsuppressed HIV viral loads, rectal bleeding (25 vs. 18%) and purulent or bloody stools (22 vs. 14%) were even more prevalent (9). The patient in our case report was hospitalized for 2 months, while non-complicated patients with Mpox typically stay for a maximum of 2 weeks. In addition, during the course of her illness, our patient developed a fulminant form of Mpox with more disseminated pustular eruptions that coalesced into large bullae and ruptured, leaving necrotic ulcers. The complication of septicemia, attributed to the secondary bacterial infection of the skin lesions and immunosuppression observed in our case, was also reported in a report from a global series of Mpox in patients with advanced HIV (8). Severe genital ulcers, characterized by painful vulvar edema, bacterial superinfection, and pus discharge, are common findings in these types of cases. Despite the lack of evidence, it is also believed that the prolonged duration and greater number of skin lesions in individuals with HIV could be associated with a longer period of infectivity.

### Strength

This case was reported by dermatologists with a clear understanding of the disease and its skin manifestations, providing a valuable reference for future cases, especially as the outbreak continues in various parts of the world, particularly in Africa.

### Limitations

Despite the clinical staging of this case as advanced HIV, the lack of viral load and CD4 count data is considered a limitation in substantiating the degree of immunosuppression due to her HIV infection and its influence on the clinical manifestation of Mpox.

## Conclusion

HIV coinfection can significantly worsen Mpox outcomes, leading to more severe clinical complications, prolonged illness, and an increased risk of secondary infections. Routine HIV screening should be implemented for all Mpox patients, and clinicians must be aware of the potential complications in coinfected individuals. Similarly, Mpox should be ruled out in HIV patients with suspicious skin lesions to ensure timely diagnosis and management.

## Data Availability

The datasets presented in this study can be found in online repositories. The names of the repository/repositories and accession number(s) can be found at.
